# First Molecular Detection of *Babesia gibsoni* in Stray Dogs from Thailand

**DOI:** 10.3390/pathogens10060639

**Published:** 2021-05-22

**Authors:** Thom Do, Ruttayaporn Ngasaman, Vannarat Saechan, Opal Pitaksakulrat, Mingming Liu, Xuenan Xuan, Tawin Inpankaew

**Affiliations:** 1 Department of Parasitology, Faculty of Veterinary Medicine, Kasetsart University, Bangkok 10900, Thailand; dothanhthom.t@ku.th; 2 Faculty of Veterinary Science, Prince of Songkla University, Songkhla 90110, Thailand; ruttayaporn.n@psu.ac.th (R.N.); vannarat.s@psu.ac.th (V.S.); 3 Department of Parasitology, Faculty of Medicine, Khon Kaen University, Khon Kaen 40000, Thailand; opalpi@kku.ac.th; 4 National Research Center for Protozoan Diseases, Obihiro University of Agriculture and Veterinary Medicine, Obihiro, Hokkaido 080-8555, Japan; lmm_2010@hotmail.com

**Keywords:** molecular detection, vector-borne, *Babesia gibsoni*, stray dogs, Thailand

## Abstract

In southern Thailand, the increasingly growing population of stray dogs is a concern to public health and environmental safety because of the lack of medical attention and control. More importantly, these animals are considered reservoirs for many zoonotic pathogens. The objective of this study was to molecularly detect canine vector-borne pathogens, and to perform genetic characterization of *Babesia gibsoni* present in stray dogs from southern Thailand. Blood samples were collected from 174 stray dogs in two provinces (Songkhla and Narathiwat) in southern Thailand. PCR analyses were executed using specific primers based on the *Babesia* spp. 18S rRNA gene, *Babesia gibsoni* Internal transcribed spacer 1 (ITS1) region, *Ehrlichia canis* citrate synthase (gltA) gene, *Hepatozoon* spp. 18S rRNA gene and *Anaplasma platys* heat shock protein (groEL) gene. The most common canine vector-borne pathogen found infecting stray dogs in this study was *Hepatozoon canis* (24.7%) followed by *A. platys* (14.9%), *Babesia vogeli* (8.0%), *B. gibsoni* (6.3%), and *E. canis* (1.72%). Concurrent infection with more than one pathogen occurred in 72 cases. Phylogenetic analysis based on the ITS1 region and 18S rRNA gene revealed that the *B. gibsoni* isolates from this study shared a large proportion of their identities with each other and with other reported *B. gibsoni* genotypes from Asia. This study highlights the molecular detection of *B. gibsoni* in dogs in Thailand for the first time and presents the genetic characterization by sequencing the ITS1 region and 18S rRNA gene of *B. gibsoni* from Thailand. Follow-up studies are needed to elucidate the origin, distribution, and vectors of *B. gibsoni* parasites circulating in dogs in Thailand, as well as to determine to what extent dogs are important reservoir hosts for zoonotic canine vector-borne disease infection in the studied area.

## 1. Introduction

Canine vector-borne diseases (CVBDs) including ehrlichiosis, anaplasmosis, babesiosis, and hepatozoonosis are common infectious diseases in dogs caused by various bacteria and protozoa [1,2,3,4]. *Anaplasma* and *Ehrlichia* are obligate intracellular bacteria belonging to the family Anaplasmataceae [5], and are mostly detected in canids in tropical and subtropical areas [6,7]. Of the common *Ehrlichia* species, *Ehrlichia canis* is the etiologically important agent of canine monocytic ehrlichiosis [8]. *Anaplasma phagocytophilum* and *Anaplasma platys* have been documented as the main causative agents of canine anaplasmosis in temperate zones and canine cyclic thrombocytopenia in the tropical area, respectively [7,8]. In addition, of the recently emerged protozoan genera, *Babesia* and *Hepatozoon* are among the most widespread apicomplexan protozoan parasites causing severe diseases ranging from subclinical mild anemia to multiple organ failure, and sometimes death in infected dogs [9]. Specifically, at least four species of *Babesia* (*Babesia gibsoni*, *Babesia canis*, *Babesia rossi*, and *Babesia vogeli*) and two *Hepatozoon* species (*Hepatozoon canis* and *Hepatozoon americanum*) are agents of canine babesiosis and hepatozoonosis, respectively [5]. Most of the earlier mentioned canine vector-borne pathogens (CVBPs) can be transmitted to other dogs by tick bites, blood transfusion, or dog fighting, except for *Hepatozoon*, which is transmitted primarily through the ingestion of ticks containing mature *H. canis* oocysts [3,10].

*Babesia gibsoni* is found in almost all parts of Asia and is considered a notable threat to canine health, causing the acute form of babesiosis in dogs, typically related to fever, anemia, thrombocytopenia, splenomegaly, and hepatomegaly [11]. In chronic infections, the dogs commonly remain asymptomatic carriers [12]. Genetic characterization and phylogenetic analysis of the 18S rRNA gene demonstrated that there are three genotypically different small *Babesia* of canines [13] including *B. gibsoni sensu stricto* [14], *Babesia microti-type* [15], and *Babesia conradae* [16]. Moreover, the Internal transcribed spacer 1 (ITS1) region is often used to determine the divergence among species and even among strains of canine *Babesia* [17]. The high degree of variation in the ITS1 region is commonly employed to genetically differentiate the subspecies level of *Babesia* spp., which 18S rRNA has failed to demonstrate [17]. Specifically, intraspecific variation within the ITS1 region has already been reported in isolates of three subspecies of *B. canis* [17]. As for the *B. gibsoni* Asian genotype, limited studies have been carried out using partial sequence of the ITS1 region for phylogenetic placements of different isolates and to study the intraspecific genetic variability between isolates [18].

Recently, several programs of sterilization under the Thai government for the restriction of stray animal population have been frequently conducted. The epidemiology and clinical importance of *B. gibsoni* infections in Thailand are not well understood, and limited information is available on genetic characterization of *B. gibsoni* of dogs in Thailand. The present study was designed to determine the CVBPs present in stray dogs from southern Thailand, using molecular techniques and to further characterize *B. gibsoni*, using the ITS1 region.

## 2. Results

### 2.1. Occurrence of Single and Co-Infections

The PCR-based results showed that the occurrence of CVBPs in stray dog blood samples collected from Songkhla and Narathiwat provinces was 43.1% (75/174). The most common CVBP found infecting stray dogs in this study was *H. canis* (24.7%) followed by *A. platys* (14.9%), *B. vogeli* (8.0%), *B. gibsoni* (6.3%), and *E. canis* (1.7%). Co-infections were also present in this study. Infection with *A. platys* and *H. canis* (8.0%, 14/174) was the most prevalent concurrent infection, and only one sample (0.6%, 1/174) was found positive for three pathogens in the screened population (Table 1).

### 2.2. Sequence Analysis

The representative sequences of CVBPs detected in this study were submitted to Genbank under accession numbers MW404321–MW404324 (*A. platys*), MW404325–MW 404327 (*E. canis*), MW402988–MW402992 (*H. canis*), MW403069–MW403073 (*B. vogeli*), MW403495–MW403499 (*B. gibsoni*–18S rRNA gene), and MW403987–MW403991 (*B. gibsoni*–ITS1 region). Subsequently, the obtained sequences of CVBPs in the present study subjected to BLAST analysis were found to share 100% identity with the published sequences from Genbank (Table 2).

### 2.3. Phylogenetic Analysis of B. gibsoni Using the ITS1 Region and 18S rRNA Sequences

**Figure 1:**
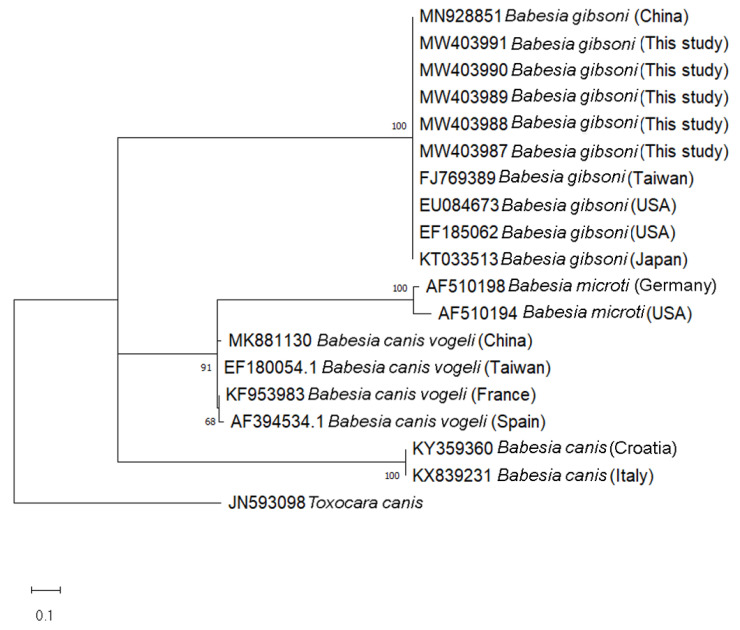
Phylogenetic tree of *Babesia gibsoni* based on the 254 bp-fragment of the ITS1 region using Maximum Likelihood method (Kimura two-parameter model). Numbers at the nodes represent percentage occurrences clades based on 1000 bootstrap replication of data. The sequence of *Toxocara canis* (JN593098) was used as outgroup.

**Figure 2:**
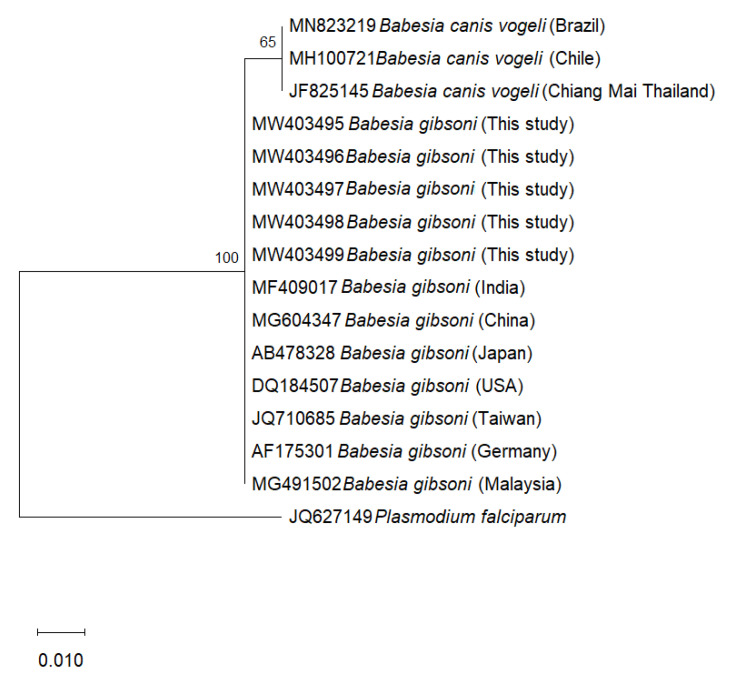
Phylogenetic tree of *Babesia gibsoni* based on the 18S rRNA region using Maximum Likelihood method (Kimura two-parameter model). Numbers at the nodes represent percentage occurrences clades based on 1000 bootstrap replication of data. The sequence of *Plasmodium falciparum* (JQ627149) was used as outgroup.

Phylogenetic trees of *B. gibsoni* were constructed based on the 254 bp-fragments of the ITS1 region and 208 bp-fragments of the 18S rRNA gene from dogs in this study, with the corresponding available database isolates using Maximum Likelihood method with Kimura two-parameter model. In the representing tree, *Toxocara canis* and *Plasmodium falciparum* were used as outgroup species to root the tree, respectively. In the ITS1-based phylogenetic tree, the isolates of each considered species including *B. gibsoni*, *B. vogeli*, *B. microti*, and *B. canis* formed separate clades with high bootstrap support (100%) (Figure 1). In the 18S rRNA- based phylogenetic tree, all the *B. gibsoni* showed low variability between the sequences obtained from this study and those from other geographic regions, including Malaysia, Japan, China, India, Taiwan, Germany and Asian genotype USA (Figure 2).

## 3. Discussion

Stray dogs are considered a major concern to the public and the environment [19] since they are not given medical care and are reservoirs for various infectious diseases, including canine vector-borne pathogens [20]. The attempt of reducing the population to prevent some infectious diseases in stray dogs is increasing in the south of Thailand, wherein a project of rabies vaccination prophylaxis and sterilization under the management of the provincial Livestock Development Department and Faculty of Veterinary Science, Prince of Songkla University was conducted. Simultaneously, a study of CVBPs detection by using molecular has been established to report the status of these pathogens circulating in the animals in this area. Our results show that CVBPs including *A.platys*, *E. canis*, *H. canis*, *B. vogeli*, and *B. gibsoni* are endemic in the Songkhla and Narathiwat provinces.

The occurrence of CVBPs were frequent in stray dogs in studied areas (43.1%, 75/174), of which *H. canis*, the causative agent of canine hepatozoonosis, was the most PCR-detected intracellular protozoan parasite (24.7%, 43/174) contracted by canine. The detection rate of these pathogens varies compared to previous reports conducted in the northern areas (41.2%, 28/68) [21] and in the southern areas (43.1%, 78/181) [22]. The variability observed in this study with regard to others could be due to the difference in the number of dogs, the selection criteria, the sampling size, the geographical area, and the sampling season [6,22]. In addition, among the common bacteria infecting the dogs, *A. platys*, an obligate intracellular Anaplasmataceae bacteria, was previously known as the most prevalent tick-borne bacteria in canids in tropical and subtropical areas in several reports [6]. The *Anaplasma platys* detection rate (14.9%) from our study showed that it was the most common bacteria found in stray dogs, which reinforced the mentioned statement. Concurrent infections with two or more CVBPs were identified in the studied dogs. This was determined in 21 individuals (12.06%), of which the most prevalent co-infection pattern was *H. canis* and *A. platys* (8%, 14/174) (Table 1). The occurrence of co-infection may cause greater pathogenicity whereby greater variable signs were exhibited by the affected dogs, resulting in a more challenging diagnosis. Our finding is in agreement with previous statements on the importance of testing for more than one CVBP [3,6].

**Figure 3:**
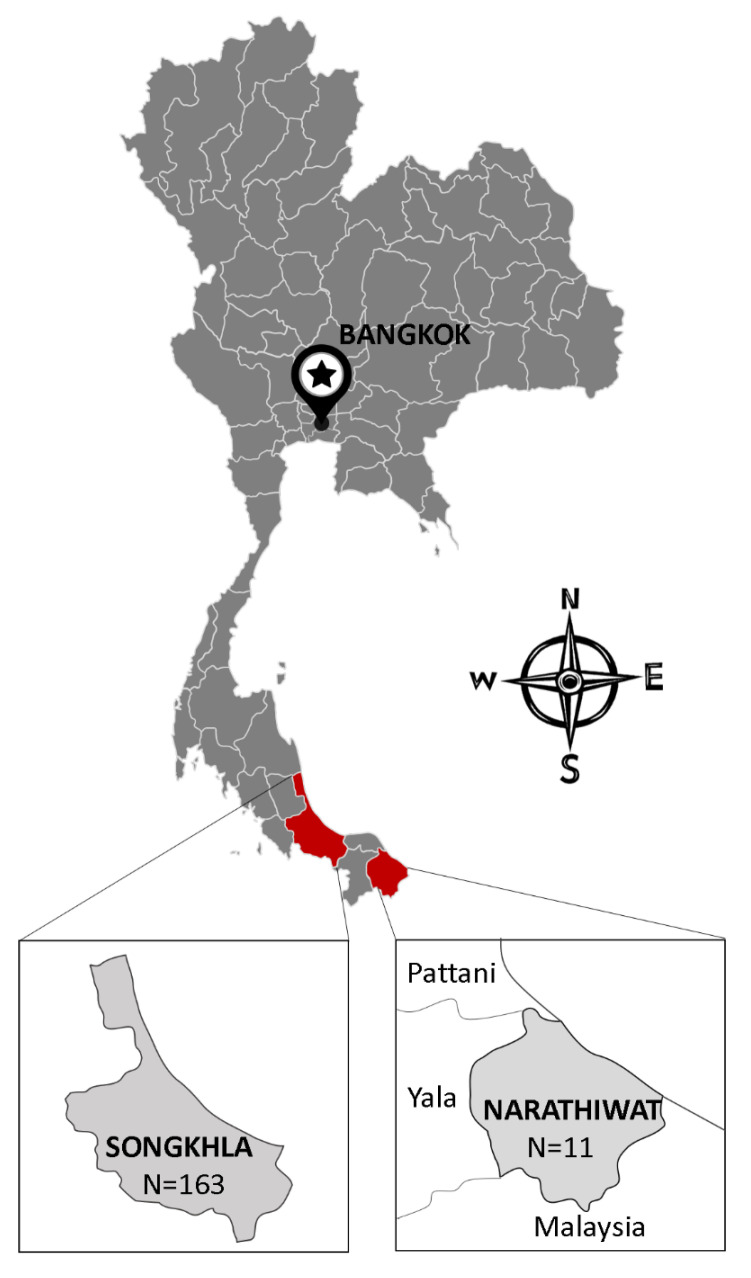
Map of Songkhla and Narathiwat province in Thailand where the dog blood samples were collected.

The present study provides the first molecular evidence of *B. gibsoni* infection detected in 11 dog blood samples. This finding is important in molecular phylogenetic studies by contributing to the literature about *Babesia* epidemiology in Thailand. *B. gibsoni* have been previously documented from dogs in different countries in Asia such as Malaysia [23], Philippines [24], China [25], and Japan [26]. The most common *Babesia* species found in dogs in Thailand are *B. canis* [27] and *B. vogeli* [21,28,29]. Ticks have been considered as the main vector of *Babesia* transmission [30], in which *Rhipicephalus Sanguineus* ticks is the most common tick species in Thailand have been reportedly as carriers of *B. canis* and *B. vogeli* [29,31]. The transmission of *B. gibsoni* through tick species found in dogs in the studied area should be taken into account in further research in order to overcome the limitation of the present investigation as the lack of the pathogen detection in ticks. Furthermore, other ways for CVBPs to spread include blood transfusions or dog fighting [3,10]. Specifically, stray dogs with their roaming behavior might spread ticks from one place to another, thereby playing an important role in CVBP spreading [6]. In the current study, sampling was conducted in the Narathiwat province and the Songkhla province. Interestingly, all samples from Narathiwat (11 samples) were positive with *B. gibsoni*, while there were no *B. gibsoni*-positive cases found in Songkha. It is found that Narathiwat shares a long border with Malaysia (Figure 3), which had reported *B. gibsoni* infection in dogs and their ticks previously [23,32,33]. Thus, this more likely deduces that the detection of *B. gibsoni* in stray dogs in the Narathiwat province might have been due to the spreading of *B. gibsoni*-infected dogs or ticks from Malaysia areas [32,33]. The attempt to analyze and compare the phylogenetic of *B. gibsoni* isolates from this study and those from Malaysia revealed that they shared a 100% identity with each other based on the 18S rRNA gene (Figure 2). However, a 100% identity in the 18S rRNA gene might not accurately reflect genetic diversity between isolates due to its low sensitivity compared to the ITS1 region, as shown in previous reports [17]. Upon phylogenetic analysis of ITS1 sequences, eleven isolates were confirmed as *B. gibsoni* and representative isolates clustered together in the *B. gibsoni* clade, with other reported *B. gibsoni* Asian genotype isolates from Japan, Taiwan, China, and the USA. The *B. gibsoni* Asian genotype isolates clustered away from the other *Babesia* spp. The genetic characterization of the ITS1 region of *B. gibsoni* from Thailand reinforced the ITS1 region as a useful genetic marker to study the genetic divergence, evolution, and relationship among *B. gibsoni* isolates. Moreover, the attempt of aligning the ITS1-based sequences of *B. gibsoni* from this study with that of Malaysia failed since the ITS1-based isolates of *B. gibsoni* are not available in Malaysia. Further investigation of *B. gibsoni* molecular characteristic should be conducted based on this genetic region to demonstrate the mentioned hypothesis. By sharing the common environment with humans and other domestic animals, the finding of *B. gibsoni* in dogs from the current study should alert Thai people about the risk of CVBPs infection transmitted from these stray animals to their pets. Therefore, a survey of the evaluation of the risk factors associated with *B. gibsoni* infection in dogs in this area should also be considered to overcome the limitation of the current study and to develop effective prevention and control strategies to minimize infections by CVBPs.

## 4. Materials and Methods

### 4.1. Ethical Consent

All the procedures were conducted according to ethical guidelines for the use of animal samples approved by the ethics committee and decision board of Prince of Songkla University (No. 416/2017) and Obihiro University of Agriculture and Veterinary Medicine (Permit for animal experiment: 290131; DNA experiment: 1724-2).

### 4.2. Study Area and Sample Collection

The sampling was conducted in the Narathiwat provinces (n = 11) and Songkhla (n = 163), located in the southern part of Thailand (Figure 2). The blood samples were collected from stray dogs under the sterilization services program between September 2014 and December 2015. Each dog was humanely restrained and at least 2 mL of blood sample was obtained from the cephalic vein into a vacutainer blood collection tube containing ethylenediamine tetra-acid (EDTA), performed by a qualified veterinary technician. Subsequently, blood samples were stored in a freezer (−20 °C) at the Faculty of Veterinary Science, Prince of Songkla University, Songkhla, Thailand, until retrieval for further laboratory investigations.

### 4.3. DNA Extraction and Molecular Detection

The genomic DNA was extracted using the Genomic DNA Mini Kit (Blood/Cultured cell) (Geneaid Biotech Ltd., New Taipei City, Taiwan) following the manufacturer’s instructions. Subsequently, conventional PCR was employed to identify the presence of DNA of *Hepatozoon* spp., *Babesia* spp., *A. platys*, and *E. canis* with specific primer sets (Table 3). The amplifications were performed in a 25 µL reaction mixture encompassed by distilled deionized water, 3 µL of template DNA (10–80 ng/µL), 10 pmol of each primer, 250 µM of each deoxynucleotide triphosphate, 2 µL of 10X Ex Taq buffer, and 1 unit of Ex Taq DNA polymerase (Takara Bio, Kyoto, Japan). Amplifications were performed using MyCycler Thermal Cycler (Bio-Rad, Hercules, CA, USA) under the conditions described in Table 3. Negative controls (distilled deionized water) and positive controls (DNA of each pathogen) were used in each PCR reaction. The PCR products were checked by electrophoresis in 1.5% agarose gel (LE agarose, Thermo Fisher Scientific, Waltham, MA, USA) and TAE (Tris-acetate-EDTA) buffer.

### 4.4. Sequence and Phylogenetic Analysis

The positive amplicons were snipped from the gel and purified using QIAquick Gel Extraction Kit (QIAGEN GmbH, Hilden, Germany) according to the manufacturer’s instructions. Subsequently, the nucleotide sequences of the target DNA fragments from both directions were determined using an ABI PRISM 3100 Genetic Analyzer (Applied Biosystems, Waltham, MA, USA). After obtaining the sequence results, the sequences were compared with published isolates using the Basic Local Alignment Search Tool (BLAST) of the U.S. National Center for Biotechnology Information (https://blast.ncbi.nlm.nih.gov/Blast.cgi, accessed on 17 May 2021) and alignment was achieved using the BioEdit program version 7.5.2 (https://bioedit.software.informer.com/, accessed on 17 May 2021).

The genetic relationship of *Babesia gibsoni* based on the ITS1 region and the 18S rRNA gene obtained in the Narathiwat province in the present study, and that from other regions of the world, was determined by phylogenetic analyses using the MEGA version X program (accessed on 17 May 2021) [39]. The maximum-likelihood method with Kimura two-parameter model was employed to construct the phylogenetic trees. Bootstrap analysis with 1000 replications was set to estimate the reliability of the branching patterns of the trees.

### 4.5. Statistical Analysis

The detection rate and confidence intervals (95%) for each species were calculated using the R software [40].

## 5. Conclusions

The present study was the first to record and characterize the phylogeny of *B. gibsoni* based on the ITS1 region and the 18S rRNA gene in dogs in southern Thailand. Further survey with additional samples from dogs and their tick vectors should be conducted to evaluate the risk factors associated with CVBPs infection in dogs in these areas to prevent and minimize the infections.

## Figures and Tables

**Table 1:** Occurrence of tick-borne pathogens in stray dogs from Songkhla and Narathiwat provinces, Thailand.

Pathogen	No. Positive (N = 174)	Detection Rate (95% CI)
Single infection		
*Anaplasma platys*	10	5.7 (2.26–9.15)
*Ehrlichia canis*	1	0.6 (0–1.75)
*Hepatozoon canis*	23	13.2 (8.17–18.23)
*Babesia gibsoni*	11	6.3 (2.69–9.91)
*Babesia vogeli*	9	5.2 (1.9–8.49)
Mixed infection		
*Anaplasma platys + Hepatozoon canis*	14	8.0 (3.96–12.03)
*Anaplasma platys + Babesia vogeli*	1	0.6 (0–1.75)
*Ehrlichia canis + Hepatozoon canis*	1	0.6 (0–1.75)
*Hepatozoon canis + Babesia vogeli*	4	2.3 (0–4.53)
*Anaplasma platys + Ehrlichia canis+ Hepatozoon canis*	1	0.6 (0–1.75)
Total positive	75	43.1 (35.74–50.46)
Negative samples	99	56.9 (49.64–64.26)

**Table 2:** Representative sequences of canine vector-borne pathogens detected in the study.

No.	Species	Gene	Length (Base-Pair)	Accession no. (Submitted)	Accession no. (Reference)	Query Cover (%)	Percent Identity (%)
1	*A. platys*	groEL	694	MW404321–MW404324	KU765205; KY425417	100	100
2	*E. canis*	gltA	1249	MW404325–MW 404327	KU765198; CP025749	100	100
3	*H. canis*	18S rRNA	666	MW402988–MW402992	KU765202; MK091085	100	100
4	*B. vogeli*	18S rRNA	208	MW403069–MW403073	MT386936; MT012237	100	100
5	*B. gibsoni*	18S rRNA	208	MW403495–MW403499	MN134517; MG604547	100	100
6	*B. gibsoni*	ITS1	254	MW403987–MW403991	MN928851; KP666153	100	100

Abbreviations: groEL: heat shock protein gene, gltA: citrate synthase gene, ITS1: Internal transcribed spacer 1.

**Table 3:** Sequences of primer sets used for the detection of canine vector-borne pathogens.

Pathogen (Target Gene)	Oligonucleotide Sequences (5′→3′)	Product Size (bp)	Annealing Temp (°C)	Reference
*Babesia* spp. (18S rRNA)	F: GCATTTAGCGATGGACCATTCAAG R: CCTGTATTGTTATTTCTTGTCACTACCTC	208	60	[34]
*Babesia gibsoni* (ITS1)	F: ACATTGAAACTTGTCGAGCTGCG R: AGATCCCGCACCCAGCCAC	254	60	[35]
*Ehrlichia canis* (gltA)	F: TTATCTGTTTATGTTATATAAGC R: CAGTACCTATGCATATCAATCC	1372	53	[36]
*Hepatozoon* spp. (18S rRNA)	F: ATACATGAGCAAAATCTCAAC R: CTTATTATTCCATGCTGCAG	666	57	[37]
*Anaplasma platys* (groEL)	F: AAGGCGAAAGAAGCAGTCTTA R: CATAGTCTGAAGTGGAGGAC	724	58	[38]

Abbreviations: F: Forward, R: Reverse, groEL: heat shock protein gene, gltA: citrate synthase gene, ITS1: Internal transcribed spacer 1.

## Data Availability

Data are contained within the article.

## References

[1] Cardoso L., Oliveira A.C., Granada S., Nachum-Biala Y., Gilad M., Lopes A.P., Sousa S.R., Vilhena H., Baneth G. (2016). Molecular investigation of tick-borne pathogens in dogs from Luanda, Angola. Parasit. Vectors.

[2] Foglia M.V., Cappiello S., Oliva G. (2006). Tick-transmitted diseases in dogs: Clinicopathological findings. Parassitologia.

[3] Inpankaew T., Hii S.F., Chimnoi W., Traub R.J. (2016). Canine vector-borne pathogens in semi-domesticated dogs residing in northern Cambodia. Parasit. Vectors.

[4] Kaewmongkol G., Lukkana N., Yangtara S., Kaewmongkol S., Thengchaisri N., Sirinarumitr T., Jittapalapong S., Fenwick S.G. (2017). Association of *Ehrlichia canis*, Hemotropic *Mycoplasma* spp. and Anaplasma platys and severe anemia in dogs in Thailand. Vet. Microbiol..

[5] Ogbu K.I., Olaolu O.S., Ochai S.O., Tion M.T. (2018). A review of some tick-borne pathogens of dogs. J. Anim. Sci. Vet. Med..

[6] Galay R.L., Manalo A.A.L., Dolores S.L.D., Aguilar I.P.M., Sandalo K.A.C., Cruz K.B., Divina B.P., Andoh M., Masatani T., Tanaka T. (2018). Molecular detection of tick-borne pathogens in canine population and *Rhipicephalus sanguineus* (sensu lato) ticks from southern Metro Manila and Laguna, Philippines. Parasit. Vectors.

[7] Little S.E. (2010). Ehrlichiosis and anaplasmosis in dogs and cats. Vet. Clin. Small Anim. Pract..

[8] Huggins L.G., Koehler A.V., Ng-Nguyen D., Wilcox S., Schunack B., Inpankaew T., Traub R.J. (2019). Assessment of a metabarcoding approach for the characterisation of vector-borne bacteria in canines from Bangkok, Thailand. Parasit. Vectors.

[9] Matijatko V., Torti M., Schetters T.P. (2012). Canine babesiosis in Europe: How many diseases?. Trends Parasitol..

[10] Baneth G.A.D., Samish M., Alekseev E., Aroch I., Shkap V. (2001). Transmission of *Hepatozoon canis* to dogs by naturally-fed or percutaneously-injected *Rhipicephalus sanguineus* ticks. J. Parasitol..

[11] Mandal M., Banerjee P.S., Kumar S., Garg R., Ram H., Raina O.K. (2016). Development of recombinant BgP12 based enzyme linked immunosorbent assays for serodiagnosis of *Babesia gibsoni* infection in dogs. Vet. Immunol. Immunopathol..

[12] Conrad P., Thomford J., Yamane I., Whiting J., Bosma L., Uno T., Holshuh H.J., Shelly S. (1991). Hemolytic anemia caused by *Babesia gibsoni* infection in dogs. J. Am. Vet. Med. Assoc..

[13] Kjemtrup A.M., Kocan A.A., Whitworth L., Meinkoth J., Birkenheuer A.J., Cummings J., Boudreaux M.K., Stockham S.L., Irizarry-Rovira A., Conrad P.A. (2000). There are at least three genetically distinct small piroplasms from dogs. Int. J. Parasitol..

[14] Zahler M., Rinder H., Zweygarth E., Fukata T., Maede Y., Schein E., Gothe R. (2002). Babesia gibsoni of dogs from North America and Asia belong to different species. Parasitology.

[15] García A.T.C. (2006). Piroplasma infection in dogs in northern Spain. Vet. Parasitol..

[16] Kjemtrup A.M., Conrad P.A. (2006). A review of the small canine piroplasms from California: *Babesia conradae* in the literature. Vet. Parasitol..

[17] Zahler M., Schein E., Rinder H., Gothe R. (1998). Characteristic genotypes discriminate between *Babesia canis* isolates of differing vector specificity and pathogenicity to dogs. Parasitol. Res..

[18] Bostrom B., Wolf C., Greene C., Peterson D.S. (2008). Sequence conservation in the rRNA first internal transcribed spacer region of *Babesia gibsoni* genotype Asia isolates. Vet. Parasitol..

[19] Toukhsati S.R., Phillips C.J.C., Podberscek A.L., Coleman G.J. (2012). Semi-ownership and sterilisation of cats and dogs in Thailand. Animals.

[20] Abd Rani P.A.M., Irwin P.J., Coleman G.T., Gatne M., Traub R.J. (2011). A survey of canine tick-borne diseases in India. Parasit. Vectors.

[21] Buddhachat K., Meerod T., Pradit W., Siengdee P., Chomdej S., Nganvongpanit K. (2020). Simultaneous differential detection of canine blood parasites: Multiplex high-resolution melting analysis (mHRM). Ticks Tick. Borne. Dis..

[22] Liu M., Ruttayaporn N., Saechan V., Jirapattharasate C., Vudriko P., Moumouni P.F.A., Cao S., Inpankaew T., Ybañez A.P., Suzuki H. (2016). Molecular survey of canine vector-borne diseases in stray dogs in Thailand. Parasitol. Int..

[23] Prakash B.K., Low V.L., Vinnie-Siow W.Y., Tan T.K., Lim Y.A.-L., Morvarid A.R., AbuBakar S., Sofian-Azirun M. (2018). Detection of *Babesia* spp. in dogs and their ticks from Peninsular Malaysia: Emphasis on *Babesia gibsoni* and *Babesia vogeli* infections in *Rhipicephalus sanguineus* sensu lato (Acari: Ixodidae). J. Med. Entomol..

[24] Cruz-Flores M.J., Garcia Claveria F., Verdida R., Xuan X., Igarashi I. (2008). First detection of *Babesia gibsoni* infection in Philippine stray dogs by immunochromatographic test (ICT). Vet. Arh..

[25] Zheng W., Liu M., Moumouni P.F.A., Liu X., Efstratiou A., Liu Z., Liu Y., Tao H., Guo H., Wang G. (2017). First molecular detection of tick-borne pathogens in dogs from Jiangxi, China. J. Vet. Med. Sci..

[26] Miyama T., Sakata Y., Shimada Y., Ogino S., Watanabe M., Itamoto K., Okuda M., Verdida R.A., Xuan X., Nagasawa H. (2005). Epidemiological survey of *Babesia gibsoni* infection in dogs in eastern Japan. J. Vet. Med. Sci..

[27] Rucksaken R., Maneeruttanarungroj C., Maswanna T., Sussadee M., Kanbutra P. (2019). Comparison of conventional polymerase chain reaction and routine blood smear for the detection of *Babesia canis*, *Hepatozoon canis*, *Ehrlichia canis*, and *Anaplasma platys* in Buriram Province, Thailand. Vet. World.

[28] Piratae S., Pimpjong K., Vaisusuk K., Chatan W. (2015). Molecular detection of *Ehrlichia canis*, *Hepatozoon canis* and *Babesia canis vogeli* in stray dogs in Mahasarakham province, Thailand. Ann. Parasitol..

[29] Colella V., Nguyen V.L., Tan D.Y., Lu N., Fang F., Zhijuan Y., Wang J., Liu X., Chen X., Dong J. (2020). Zoonotic vectorborne pathogens and ectoparasites of dogs and cats in Eastern and Southeast Asia. Emerg. Infect. Dis..

[30] Gray J.S., Estrada-Peña A., Zintl A. (2019). Vectors of babesiosis. Annu. Rev. Entomol..

[31] Do T., Phoosangwalthong P., Kamyingkird K., Kengradomkij C., Chimnoi W., Inpankaew T. (2021). Molecular Detection of Tick-Borne Pathogens in Stray Dogs and *Rhipicephalus sanguineus* sensu lato Ticks from Bangkok, Thailand. Pathogens.

[32] Rajamanickam C., Wiesenhutter E., Zin F.M., Hamid J. (1985). The incidence of canine haematozoa in Peninsular Malaysia. Vet. Parasitol..

[33] Mokhtar A.S., Lim S.F., Tay S.T. (2013). Research Note Molecular detection of *Anaplasma platys* and *Babesia gibsoni* in dogs in Malaysia. Trop Biomed.

[34] Kordick S.K., Breitschwerdt E.B., Hegarty B.C., Southwick K.L., Colitz C.M., Hancock S.I., Bradley J.M., Rumbough R., Mcpherson J.T., MacCormack J.N. (1999). Coinfection with multiple tick-borne pathogens in a Walker Hound kennel in North Carolina. J. Clin. Microbiol..

[35] Mandal M., Banerjee P.S., Garg R., Ram H., Kundu K., Kumar S., Ravi Kumar G.V.P.P.S. (2014). Genetic characterization and phylogenetic relationships based on 18S rRNA and ITS1 region of small form of canine *Babesia* spp. from India. Infect. Genet. Evol..

[36] Inokuma H., Brouqui P., Drancourt M., Raoult D. (2001). Citrate Synthase Gene Sequence: A New Tool for Phylogenetic Analysis and Identification ofEhrlichia. J. Clin. Microbiol..

[37] Inokuma H., Okuda M., Ohno K., Shimoda K., Onishi T. (2002). Analysis of the 18S rRNA gene sequence of a *Hepatozoon* detected in two Japanese dogs. Vet. Parasitol..

[38] Inokuma H., Fujii K., Okuda M., Onishi T., Beaufils J.-P., Raoult D., Brouqui P. (2002). Determination of the nucleotide sequences of heat shock operon groESL and the citrate synthase gene (gltA) of *Anaplasma* (*Ehrlichia*) *platys* for phylogenetic and diagnostic studies. Clin. Diagn. Lab. Immunol..

[39] Kumar S., Stecher G., Li M., Knyaz C., Tamura K. (2018). MEGA X: Molecular Evolutionary Genetics Analysis across Computing Platforms. Mol. Biol. Evol..

[40] Pinheiro J., Bates D., DebRoy S., Sarkar D., Team R.C. (2013). nlme: Linear and nonlinear mixed effects models. R Packag. Version.

